# Occupational stress and associated risk factors among 13,867 industrial workers in China

**DOI:** 10.3389/fpubh.2022.945902

**Published:** 2022-11-17

**Authors:** Tenglong Yan, Fang Ji, Mingli Bi, Huining Wang, Xueting Cui, Baolong Liu, Dongsheng Niu, Leilei Li, Tian Lan, Tingting Xie, Jie Wu, Jue Li, Xiaowen Ding

**Affiliations:** ^1^Beijing Institute of Occupational Disease Prevention and Treatment, Beijing, China; ^2^School of Public Health, North China University of Science and Technology, Tangshan, China; ^3^Canvard College, Beijing Technology and Business University, Beijing, China

**Keywords:** occupational stress, industrial enterprises, risk factors, workers, the new brief job stress questionnaire

## Abstract

**Objective:**

Occupational stress is a critical global public health problem. We aimed to evaluate the prevalence of occupational stress among the workers in the electricity, heat, gas, water production and supply (EHGWPS), manufacturing, and transportation industries in Beijing, China. We explored the demographic differences in occupational stress status among workers in industrial enterprises.

**Methods:**

A cross-sectional study was conducted on 13,867 workers. The self-administered New Brief Job Stress Questionnaire was used to evaluate high occupational stress status, which includes four sub-dimensions (job stressors, stress response, social support, job stressors & social support). Multiple regression and logistic regression models were used to estimate the association between high occupational stress and the four occupational stress sub-dimensions with risk factors.

**Results:**

A total of 13,867 workers were included. The prevalence of high occupational stress was 3.3% in the EHGWPS industries, 10.3% in manufacturing, and 5.8% in transportation. The prevalence of high occupational stress was higher than in the other two categories (*p* < 0.05) in manufacturing industries. Logistic regression analysis showed that male workers with lower educational status, more job experience, and working in manufacturing were vulnerable to high occupational stress. Further analysis of the four occupational stress sub-dimensions showed that male workers, older adult workers, workers with lower educational levels, and longer working time were associated with higher scores in job stressors, stress response, social support, and job stress & social support (all *p* < 0.05). Moreover, divorced or widowed workers had higher occupational stress scores.

**Conclusion:**

Male workers with lower educational levels and longer working time may have an increased risk of occupational stress.

## Introduction

Stress and depression have reached epidemic levels worldwide and have become critical global health problem concerns. There are strong evidence linking occupational activity and occupational stress to adverse health consequences. These include work absence, hypertension, cardiovascular disorders, and substance use (1–3). Occupational stress is also a major cause of mental disease, injury, and high staff turnover (4–6). Occupational stress is defined by the National Institute for Occupational Safety and Health as the stress that occurs when job demands are inconsistent with an employee's ability, employer expectations, and emotional responses (7). Globally, about three million employees have complained about occupational stress problems, and the prevalence of occupational stress varied from 30 to 52.5% (8, 9). Occupations with commonly high occupational stress risks, such as doctors and nurses, have widely reported occupational stress prevalence and risk factors (10–12). Other industries with a large number of employees (such as manufacturing or transportation enterprises) may be under-reporting the issue at high risk of occupational stress.

Employees in the manufacturing and transportation industries are distinguished from other professionals, such as doctors and nurses. Most employees are less educated, have low annual family incomes, and are at a higher risk of work stress and mental health problems, particularly those working in cities. While most studies have focused on occupational stress concerns for doctors, nurses, teachers, and other groups (12, 13), few studies have reported the prevalence of occupational stress in larger samples or compared workers in different kinds of enterprises. Evidence from epidemiological studies suggests that salary, educational status, and job role likely contribute to the development of occupational stress. Several theses on work have shown companies that use an autonomous model with a payment–reward imbalance (14), suggesting that employees in industry enterprises are a vulnerable population for occupational stress. Workers with a lower education exhibited more depressive symptoms during the coronavirus disease (COVID-19) pandemic in Istanbul and the Philippines (12, 13). Workers with lower wages have higher job insecurity or temporariness, which is significantly positively associated with higher job stress (15). Workers in these enterprises are primarily engaged in repetitive, monotonous work, which increases their risk of occupational stress. However, the prevalence of occupational stress among workers in industrial enterprises is unclear, although risk factors for occupational stress are undoubtedly present. Hence, we hypothesized that workers in these enterprises would be at a high risk of occupational stress. This study aimed to determine the prevalence of occupational stress, and compare it between different industries.

The present study conducted an extensive cross-sectional survey of 13,867 industrial workers in Beijing, China, from 2021 to 2022. We used the self-administered New Brief Job Stress Questionnaire (BJSQ) to evaluate occupational stress status. The prevalence of occupational stress and its risk factors were analyzed. This study provides evidence for a better understanding of occupational stress in industrial enterprises.

## Materials and methods

### Participants

This study used a convenience sampling method to select participants from the manufacturing industry in Beijing, China, with the enterprise as the sampling unit. Nineteen enterprises were selected, which included two electricity, heat, gas, water production, and supply (EHGWPS) enterprises, two transportation enterprises, and 15 manufacturing enterprises. Participants were selected based on the following criteria: (1) 18 years old and above; and (2) have worked for at least 12 months of in one of these enterprises. A total of 14,964 workers voluntarily participated in this study and completed the questionnaire. After excluding incomplete questionnaire, 13,867 questionnaires were valid (92.7%).

### Questionnaire

The BJSQ was used to evaluate occupational stress status in the workplace. Its reliability and validity were tested (16). The tool is a 57-item multidimensional job stress questionnaire that uses four Likert scale response options (ranging from “strongly agree” = 4 to “strongly disagree” = 1) to measure three sub-dimensions, including “job stressors,” “stress response,” and “social support.” The sub-dimension of “job stressors” adds “social support” as a new sub-dimension named “job stressors & social support.”

Higher scores indicated more obvious symptoms and severe stress. The “job stressors” scale includes the following nine factors: (1) quantitative job overload, (2) qualitative job overload, (3) physical demands, (4) interpersonal conflict, (5) poor physical environment, (6) job control, (7) skill discretion, (8) job fitness, and (9) job satisfaction. The “job stressors” scale can yield a total score ranging from 17 to 68. The “stress response” scale includes the following six indicators: (1) lack of vigor, (2) irritability, (3) fatigue, (4) anxiety, (5) depressed mood, and (6) somatic symptoms. Each participant's stress response factor yielded a score ranging from 29 to 116. The social support scale includes the following four indicators: (1) supervisor support, (2) coworker support, (3) family support, and (4) life-job satisfaction. The social support scale can yield a total score ranging from nine to 36. According to the occupational stress examination system in Japanese enterprises, participants were considered to have high occupational stress if (1) their total stress response scores were above 77, or (2) their total job stressors and social support scores were above 76, and their total stress response scores were above 63 (17).

### Data collection

This cross-sectional study was conducted on healthy workers from 19 enterprises in Beijing, China, from September 2021 to March 2022. The study was based on a self-administered survey that used a Chinese edition of the BJSQ and focused on occupational stress. Data were collected using an online questionnaire instead of face-to-face interviews due to the COVID-19 epidemic. The link to the online questionnaire was sent to workers by enterprise occupational health managers to recruit study participants.

The study protocol was approved by the Medical Ethics Committee of the Beijing Institute of Occupational Disease Prevention and Treatment. All the workers consented to participate in the study.

### Statistical analysis

After checking for completeness, the data were analyzed using SPSS (version 24.0, SPSS, Chicago, IL, USA). Continuous variables are expressed as mean ± standard deviation (*SD*), while categorical variables are expressed as numbers and percentages. Analysis of variance (ANOVA) and chi-square (χ^2^) tests were used to compare continuous and categorical variables between groups. Multiple regression models were performed to explore the association between demographic factors and job stressors scores, stress response scores, social support scores, and job stressors & social support scores. Multivariate binary logistic regression analysis was used to explore demographic factors associated with highly stressed people, and the odds ratio (*OR)* and 95% confidence interval (95% *CI*) of each factor were calculated. Job control was a crucial factor associated with position/grade, annual family income, and occupational stress. This was adjusted in the multiple regression and multivariate binary logistic regression analyses. Demographic factors such as gender (male and female), age (18–25, 26–35, 36–50, and 51–60 years), education level (middle school or below, high school, and college/university or above), marital status (never married, married, and divorced or widowed), job experience (<5, 5–9, 10–14, and ≥15 years), job control, and enterprise category (EHGWPS industries, transportation industries, and manufacturing industries) were all adjusted for in the models. Statistical significance for two-tailed *p*-values was defined as α < 0.05.

## Results

### The demographic characteristics of participants

In total, 13,867 workers participated in this study, 78.3% of whom were male workers. The mean ± SD age of the workers was (36.7 ± 8.3). Among the participants, 84.9% were between the ages of 26 and 50, 95% were less educated, and 75.9% were married. It was also observed that 13.1, 21.3, 26.3, and 39.3% of the participants had <5, 5–9, 10–14, and ≥15 years of job experience, respectively. The workers' demographic characteristics are shown in Table 1, and the categories of the 19 enterprises are shown in Supplementary Table 1.

**Table 1:** Workers' demographic characteristics (*n* = 13, 867).

**Characteristics**	**All workers** **(*n =* 13,867)**	**Workers in the EHGWPS** **industries (*n =* 273)**	**Workers in the manufacturing industries** **(*n =* 9,887)**	**Workers in the transportation industries** **(*n =* 3,707)**
**Sex**				
Male	10,863 (78.3)	231 (84.6)	8,115 (82.1)	2,517 (67.9)
Female	3,004 (21.7)	42 (15.4)	1,772 (17.9)	1,190 (32.1)
Age (years)	36.7 ± 8.3	39.4 ± 11.1	35.6 ± 7.7	39.7 ± 8.9
18~25	1,125 (8.1)	39 (14.3)	838 (8.5)	248 (6.7)
26~35	5,688 (41.0)	72 (26.4)	4,606 (46.6)	1,010 (27.2)
36~50	6,087 (43.9)	100 (36.6)	3,946 (39.9)	2,041 (55.1)
51~60	967 (7.0)	62 (22.7)	497 (5.0)	408 (11.0)
**Education**				
Middle school or below	9,946 (71.7)	159 (58.2)	6,695 (67.7)	3,092 (83.4)
High school	3,347 (24.2)	102 (37.4)	2,636 (26.7)	609 (16.4)
College/university or above	574 (4.1)	12 (4.4)	556 (5.6)	6 (0.2)
**Marital status**				
Never married	2,917 (21.1)	64 (23.4)	2,262 (22.8)	591 (15.9)
Married	10,531 (75.9)	203 (74.4)	7,422 (75.1)	2,906 (78.4)
Divorced or Widowed	419 (3.0)	6 (2.2)	203 (2.1)	210 (5.7)
**Job experience (years)**				
< 5	1,816 (13.1)	31 (11.4)	1,400 (14.2)	385 (10.4)
5~9	2,957 (21.3)	47 (17.2)	2,218 (22.4)	692 (18.7)
10~14	3,645 (26.3)	43 (15.8)	2,800 (28.3)	802 (21.6)
≥15	5,449 (39.3)	152 (55.7)	3,469 (35.1)	1,828 (49.3)

EHGWPS industries: electricity, heat, gas, and water production and supply industries.

### BJSQ scores and high occupational stress by industry categories

Descriptive statistics of each BJSQ of sub-dimension and the results of the ANVOA for each enterprise category are shown in Table 2. The manufacturing industry workers' scores of job stressors (42.94 ± 6.92), stress response (54.61 ± 15.20), and job stressors & social support (65.15 ± 9.23) were significantly higher than those of the workers in EHGWPS and transportation industries (*p* < 0.05). The manufacturing industry workers' scores for social support (22.21 ± 4.51) were significantly higher than those of transportation industry workers (21.67 ± 4.47) (*p* < 0.05). Regarding manufacturing industry workers, the scores of most BJSQ indicators were significantly higher than those of the workers in the transportation industries (*p* < 0.05). Manufacturing industry workers had significantly higher levels of qualitative job overload, interpersonal conflict, poor physical environment, job control, job satisfaction, lack of vigor, irritability, fatigue, anxiety, depressed mood, somatic symptoms, support, family support, and life-job satisfaction than EHGWPS industry workers (*p* < 0.05). EHGWPS industry workers had significantly higher indicators of quantitative job overload, interpersonal conflict, skill discretion, job fitness, and job satisfaction than transportation industry workers (*p* < 0.05).

**Table 2:** The difference in the BJSQ among three types of industrial workers.

**Indicators**	**All workers** **(*n =* 13,867)**	**Workers in the EHGWPS industries** **(*n =* 273)**	**Workers in the manufacturing industries** **(*n =* 9,887)**	**Workers in the transportation industries** **(*n =* 3,707)**	***p*** **-value**
**Job stressors**					
Quantitative job overload	8.39 ± 2.13	8.43 ± 1.73	8.67 ± 2.04	7.65 ± 2.22	< 0.001^*^^b^^c^
Qualitative job overload	10.50 ± 1.61	10.06 ± 1.76	10.38 ± 1.63	10.88 ± 1.49	< 0.001^*^^a^^b^^c^
Physical demands	3.12 ± 0.80	2.81 ± 0.73	3.10 ± 0.85	3.14 ± 0.79	< 0.001^*^^a^^b^^c^
Interpersonal conflict	5.58 ± 2.10	5.32 ± 1.94	5.77 ± 2.11	5.09 ± 2.02	< 0.001^*^^a^^c^
Poor physical environment	2.72 ± 1.08	2.45 ± 1.04	2.55 ± 1.04	2.79 ± 1.08	< 0.001^*^^a^^c^
Job control	6.71 ± 2.42	6.00 ± 1.83	6.56 ± 2.35	7.18 ± 2.56	< 0.001^*^^a^^b^^c^
Skill discretion	2.35 ± 0.98	2.39 ± 0.83	2.04 ± 0.94	2.47 ± 0.97	< 0.001^*^^b^^c^
Job fitness	1.57 ± 0.76	1.56 ± 0.68	1.37 ± 0.62	1.64 ± 0.80	< 0.001^*^^b^^c^
Job satisfaction	1.45 ± 0.76	1.37 ± 0.64	1.25 ± 0.55	1.53 ± 0.82	< 0.001^*^^a^^b^^c^
Job stressors scores	42.40 ± 6.92	40.39 ± 6.37	42.94 ± 6.92	41.12 ± 6.78	< 0.001^*^^a^^c^
**Stress response**					
Lack of vigor	7.51 ± 2.64	7.25 ± 2.33	7.70 ± 2.64	7.03 ± 2.60	< 0.001^*^^a^^c^
Irritability	5.18 ± 1.82	4.82 ± 1.61	5.33 ± 1.85	4.80 ± 1.68	< 0.001^*^^a^^c^
Fatigue	5.85 ± 2.07	5.41 ± 1.74	6.02 ± 2.11	5.43 ± 1.90	< 0.001^*^^a^^c^
Anxiety	5.34 ± 2.03	4.85 ± 1.71	5.47 ± 2.05	5.01 ± 1.93	< 0.001^*^^a^^c^
Depressed mood	9.62 ± 3.41	9.11 ± 2.98	9.96 ± 3.49	8.75 ± 3.03	< 0.001^*^^a^^c^
Somatic symptoms	19.83 ± 6.18	18.97 ± 5.45	20.11 ± 6.27	19.12 ± 5.91	< 0.001^*^^a^^c^
Stress response scores	53.33 ± 15.01	50.40 ± 13.05	54.61 ± 15.20	50.15 ± 14.10	< 0.001^*^^a^^c^
**Social support**					
Supervisor support	8.13 ± 1.85	8.03 ± 1.83	8.14 ± 1.86	8.11 ± 1.83	0.507
Coworker	7.25 ± 1.70	6.97 ± 1.66	7.29 ± 1.70	7.17 ± 1.70	< 0.001^*^^a^^c^
Family support	6.67 ± 1.84	6.56 ± 1.77	6.78 ± 1.83	6.38 ± 1.82	< 0.001^*^^a^^c^
Life-job satisfaction	3.22 ± 1.17	3.00 ± 1.06	2.85 ± 0.99	3.37 ± 1.20	< 0.001^*^^a^^b^^c^
Social support scores	22.05 ± 4.50	21.56 ± 4.30	22.21 ± 4.51	21.67 ± 4.47	< 0.001^*^^c^
**Job stressors and Social support**	64.45 ± 9.24	61.95 ± 9.03	65.15 ± 9.23	62.79 ± 9.03	< 0.001^*^^a^^c^
High stressed people					< 0.001^*^^a^^c^
Yes	1, 237 (8.9)	9 (3.3)	1, 014 (10.3)	214 (5.8)	
No	12, 630 (91.1)	264 (96.7)	8, 873 (89.7)	3, 493 (94.2)	

* *p* < 0.05;

a *p* < 0.05, EHGWPS industries compared with manufacturing industries;

b *p* < 0.05, EHGWPS industries compared with transportation industries;

c *P* < 0.05, manufacturing industries compared with transportation industries. EHGWPS industries: electricity, heat, gas, and water production and supply industries.

The prevalence of high occupational stress among participants was 8.9%. Table 2 shows the prevalence of high occupational stress among the three different industries. It was found that 10.3% of manufacturing industry workers had high occupational stress, which was significantly higher than workers in EHGWPS (3.3%) and transportation industry workers (5.8%) (*p* < 0.05).

### Influencing factors of BJSQ scores and high occupational stress status

**Figure 1:**
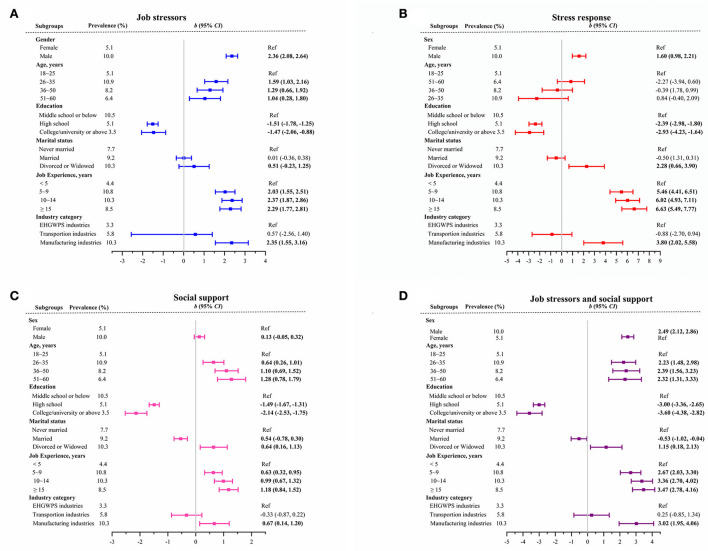
**(A–D)** Multiple linear regression random intercept model, of high occupational stress sub-dimensions.

**Figure 2:**
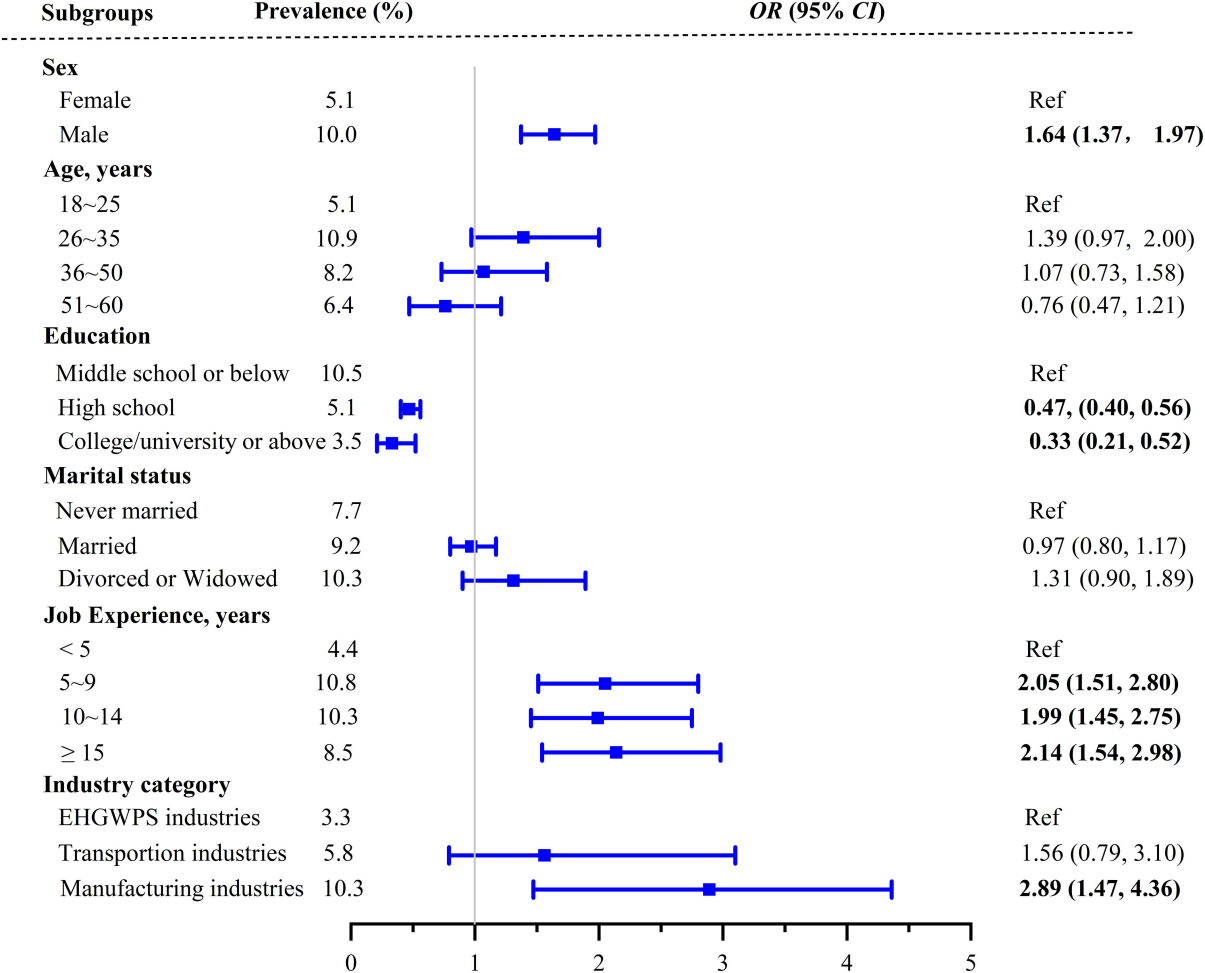
Association between factors and high occupational stress status.

Five demographic factors (sex, age, education level, marital status, and job experience) and the type of industries were the categorical variables in the multiple linear regression analysis, as shown in Figure 1. We observed a statistically positive association between several demographic factors and four sub-dimensions. Several demographic factors were also negatively associated with four sub-dimensions. For example, job stressors were positively associated with sex, age, job experience, and manufacturing enterprises (*p* < 0.05), while stress responses were negatively associated with higher education (*p* < 0.05). We observed that the manufacturing industry category was positive for all four sub-dimensions (*p* < 0.05). The relationships among high occupational stress, demographic factors, and industry category were analyzed using multivariate binary logistic regression. The results are shown in Figure 2. We observed statistically positive associations between high occupational stress and two demographic factors (including sex and job experience) and a negative association between high occupational stress status and education level (*p* < 0.05). The manufacturing industry category was positively associated with high occupational stress (*p* < 0.05).

## Discussion

In this study, we found that the prevalence of high occupational stress was 8.9% among workers in industrial enterprises; 3.3% in the EHGWPS enterprises; 10.3% in manufacturing enterprises; and 5.8% in transportation enterprises. Furthermore, the study also provided evidence that factors such as sex, education status, job experience, and industry category were associated with high occupational stress status.

In modern society, high occupational stress contributes to a series of mental and physical illnesses. It causes psychological issues, cardiovascular disease, and musculoskeletal disorders (18), costing healthcare systems approximately 300–400 million dollars per year (19). At the same time, high occupational stress is a major factor negatively affecting employees' performance, attitudes, and behaviors (20, 21). Therefore, identifying the prevalence and related factors of high occupational stress among industry workers is important to safeguard the workforce. This study supports public health efforts to provide greater protection for workers in industrial enterprises.

Current literature on occupational stress among industrial enterprise workers has not been widely reported. The available studies have primarily been conducted on occupational stress among manufacturing service and petroleum enterprises, which indicated that the prevalence of occupational stress varied from 15.8 to 72.2% among workers (22–25). The prevalence of occupational stress among electronic manufacturing service companies was reported to be 19.5 and 15.8%, according to the high strain and effort-reward imbalance models, respectively (25). Several studies have reported that occupational stress is common among nurses and doctors, two professions that are often the focus of occupational stress research. The prevalence of occupational stress among hospital staff was 45.0% (95% *CI* 24.3–67.5%) during the COVID-19 epidemic, according to a review article (26). Occupational stress prevalence was 41.2% in a cohort of Australian nurses (27). In this study, we found that the prevalence of high occupational stress was 8.9% among workers in 19 enterprises, lower than in most reports. Most studies use easier criteria for determining occupational stress, while our study only reported high occupational stress; the criteria for occupational stress were more stringent, which may explain the lower prevalence. Workers in industrial enterprises are likely to face fewer challenges than hospital employees. Their work generally has fixed and clear goals, with established methods and routines. In addition, the data of this study were from the Healthy Enterprises Construction Activities; the overall health promotion measures of these enterprises were better than those of standard enterprises.

The BJSQ scores and prevalence of occupational stress in the manufacturing industry were higher than both those of the EHGWPS and transportation industries. However, they were lower than doctors and nurses, which may be due to differences in workplace and job content. This demonstrates the necessity of identifying the specific prevalence of occupational stress in different occupations (10, 11, 13). The manufacturing industry is a typical labor-intensive industry, with many assembly line operations and repetitive mechanical labor. The working process is tedious and monotonous. Therefore, workers are vulnerable to high occupational stressors. A study has indicated that occupational stress caused by assembly line work is likely associated with changes in immune function; salivary sIgA and lysozyme are potential biomarkers (28).

To determine the factors affecting BJSQ scores among workers, we performed a multivariate analysis. The results showed that sex, age, educational status, and job experience were likely influencing factors of the four occupational stress sub-dimensions. Wang et al. reported that the prevalence of occupational stress in male electronic manufacturing service workers (19.4%) was higher than that in female workers (12.6%) (25). This may be due to the male tendency to undertake more work in industrial enterprises (29). Older adult workers with longer job experience had higher job stressors than younger workers, while the social support scores of older workers were higher than those of younger workers. Hsu HC also reported that old age was related to more work stressors, but typical psychological health (30). Workers with higher education had lower job stressors and higher social support and a lower prevalence of occupational stress. This was consistent with the studies by Assari et al. (31) and Kakemam et al. (32). Because education level is related to salary, job position/grade, and human stress-adjusting ability factors (33–35), the actual relationship between education level and occupational stress may be complex. Furthermore, the outbreak of COVID-19 has increased the frequency and duration of mask use. This can affect the humidification process of inhaled air, potentially leading to an inflammatory response of the upper respiratory tract, and increasing the risk of occupational stress (36).

To our knowledge, this is the first study to report the prevalence of occupational stress among the manufacturing, transportation, and EHGWPS industries to determine the prevalence and explore the occupational stress factors in large samples in China. The current findings suggest that the prevalence of occupational stress in these populations was low. Demographic factors, including sex, age, educational status, and job experience, were also associated with high occupational stress. In particular, workers in manufacturing enterprises were more vulnerable to occupational stress than those in other enterprises. Such workers are particularly susceptible to occupational stress, and additional prevention and health promotion efforts are needed to reduce occupational stressors (37).

This study had some limitations. Our sample of EHGWPS and transportation enterprises was low, which affects the comparability across industries. Because this was a cross-sectional study, causal inferences could be made. Further research should be conducted to determine predictors of occupational stress among industrial workers. Furthermore, a detailed assessment of related risk factors (e.g., long working hours, position, and salary) was not performed, which made it impossible to evaluate the associations between occupational stress and these factors. A more comprehensive assessment of the demographic and work-related factors should be considered in further studies. Third, despite the study's large sample size, the workers were all from a single province in China. This likely affected the extrapolation of the conclusion.

## Conclusion

The prevalence of occupational stress is not high in the EHGWPS, manufacturing, and transportation industry workers in Beijing, China, but is still worthy of attention. Workers in manufacturing enterprises with a lower education status, who were male, and who had more job experience were more vulnerable to occupational stress. Further research is needed to help improve the wellbeing of these workers and minimize poor mental health in the manufacturing and transportation industries.

## Data availability statement

The original contributions presented in the study are included in the article/Supplementary material, further inquiries can be directed to the corresponding author/s.

## Ethics statement

The studies involving human participants were reviewed and approved by the Medical Ethics Committee of the Beijing Institute of Occupational Disease Prevention and Treatment. Written informed consent for participation was not required for this study in accordance with the national legislation and the institutional requirements.

## Author contributions

Conceptualization, study design, and original draft preparation: TY and XD. Data collection, data analysis, and interpretation: TY, FJ, MB, HW, XC, TL, TX, and JW. Review and editing: BL, DN, JL, and XD. All authors have read and agreed to the published version of the manuscript.

## Conflict of interest

The authors declare that the research was conducted in the absence of any commercial or financial relationships that could be construed as a potential conflict of interest.

## Publisher's note

All claims expressed in this article are solely those of the authors and do not necessarily represent those of their affiliated organizations, or those of the publisher, the editors and the reviewers. Any product that may be evaluated in this article, or claim that may be made by its manufacturer, is not guaranteed or endorsed by the publisher.
