# Supplementary material for: Occupational stress and associated risk factors among 13,867 industrial workers in China
Source: Front Public Health. 2022 Nov 17;10:945902. doi: 10.3389/fpubh.2022.945902 (PMC9714303; doi:10.3389/fpubh.2022.945902)
Supplement: Supplementary file 1 [file Table_1.DOCX]

**Table S1.** The characteristics of participating corporations

| Corporation No. | Industry category | Number of participants |
| --- | --- | --- |
| 1 | M | 154 |
| 2 | M | 460 |
| 3 | E | 117 |
| 4 | M | 102 |
| 5 | M | 4442 |
| 6 | M | 201 |
| 7 | M | 465 |
| 8 | E | 156 |
| 9 | M | 296 |
| 10 | M | 410 |
| 11 | M | 361 |
| 12 | M | 310 |
| 13 | T | 2318 |
| 14 | M | 286 |
| 15 | M | 100 |
| 16 | M | 1372 |
| 17 | M | 470 |
| 18 | T | 1389 |
| 19 | M | 458 |
| Total |  | 13 867 |

E: Electricity, heat, gas and water production and supply industry;

M: Manufacturing industry;

T: Transportation industry;
